# Brief summary of the regulatory frameworks of regenerative medicine therapies

**DOI:** 10.3389/fphar.2024.1486812

**Published:** 2025-01-22

**Authors:** Jinah Yoon, Sukhyang Lee, Min Jung Kim, Jung-Hyun Kim

**Affiliations:** ^1^ Department of Law, Hanyang Cyber University, Seoul, Republic of Korea; ^2^ College of Pharmacy, Ajou University, Suwon, Republic of Korea; ^3^ Department of Biohealth Regulatory Science, Graduate School of Ajou University, Suwon, Republic of Korea

**Keywords:** regenerative medicine, legislation, regulatory authorities, cell therapy, gene therapy

## Abstract

The rapid advancements in regenerative medicine (RM), including cell therapies, gene therapies, tissue-engineered products, and combined RM advanced therapies, require the development of regulatory frameworks. The global landscape of regulatory frameworks presents diverse approaches to the oversight of these therapies, posing challenges in the global application of RM. This paper reviews the regulatory frameworks for RM across the United States, European Union, Japan, Canada, Australia, Taiwan, and South Korea and compares the unique features of the respective legislations.

## Introduction

Regenerative medicine is a multidisciplinary field that aims to replenish or restore defected human cells, tissues, or organs to normal function. Typically, these techniques applied as a single or combined therapies using human cells, genes and other bioengineered for the therapeutical purpose (Atala et al., 2019; Mao and Mooney, 2015). Recently, RM products have expanded to include diverse types such as cell and gene therapies, immunotherapies like CAR-T, bioengineered therapies, organoid-based regenerative medicines, exosomes, microbiomes, and xenotransplantation products that are now in clinical use (Table 1). In addition, the RM market has rapidly grown in recent years, with numerous therapeutics to treat chronic diseases, cancers, and acute diseases being approved worldwide (ASoGCTAa, 2024). As of June 2024, 32 gene therapies, including genetically modified cell therapies, have been approved (ASoGCTAa, 2024; Ilic and Liovic, 2020). Recently, CT-053 (CARsgen) was approved for myeloma in China, and Beqvez (Pfizer) was approved for hemophilia B in Canada. Additionally, 28 RNA therapies and 68 non-genetically modified cell therapies have been approved, including Amtagvi in the US for the treatment of melanoma (ASoGCTAa, 2024; U.S. Food & drug administration, 2024). Strikingly, many more gene, cell, and RNA therapies are in development, with 4,002 therapies ranging from preclinical stages to pre-legislation in the public domain. Specifically, 2,093 gene therapies, including genetically modified cell therapies like CAR-T cell therapies, are in development, accounting for 52% of gene, cell, and RNA therapies. Furthermore, 885 non-genetically modified cell therapies are in development, representing 22% of gene, cell, and RNA therapies (ASoGCTAa, 2024).

**TABLE 1:** Approved RMs in US (2020–2024.4).

Product name	Manufacturer	Date of approval	RM type	Formulation/Target
Beqbez	Pfizer	2024.04	Gene Therapy	Fidanacogene elaparvovec-dzkt/severe hemophilia B
Lenmelday	Orchard Therapeutics (Europe)	2024.03	Gene Therapy	Atidarsagene autotemcel/pre-symptomatic late infantile (PSLI), pre-symptomatic early juvenile (PSEJ) or early symptomatic early juvenile (ESEJ) metachromatic leukodystrophy (MLD)
Amtagvi	Iovance Biotherapeutics	2024.02	Cell Therapy	Lifileucel/melanoma
Lyfgenia	bluebird bio	2023.12	Gene Therapy	Lovotibeglogene autotemcel/sickle cell disease
Casgevy	Vertex Pharmaceuticals	2023.12	Gene Therapy	Exagamglogene autotemcel/beta thalassaemia and sickle cell disease
Roctavian	BioMarin Pharmaceutical	2023.06	Gene Therapy	Valoctocogene roxaparvovec-rvox/hemophilia A
Lantidra	CellTrans	2023.06	Tissue-engineered therapy	Donislecel-jujn/Type 1 diabetes
Elevidys	Sarepta Therapeutics	2023.06	Gene Therapy	Delandistrogene moxeparvovec-rokl/Duchenne muscular dystrophy (DMD)
Vyjuvek	Krystal Biotech	2023.05	Gene Therapy	Beremagene-geperpavec/dystrophic epidermolysis bullosa
Omisirge	Gamida Cell	2023.04	Cell Therapy	Omidubicel-onlv/hematologic malignancies
Adstiladrin	Ferring Pharmaceuticals	2022.12	Gene Therapy	Nadofaragene firadenovec-vncg/non-muscle invasive bladder cancer (NMIBC)
Hemgenix	CSL Behring	2022.11	Gene Therapy	Adeno-associated virus vector-based gene therapy/severe hemophilia B
Skysona	bluebird bio	2022.09	Gene Therapy	Elivaldogene autotemcel/cerebral adrenoleukodystrophy
Zynteglo	bluebird bio	2022.08	Gene Therapy	Betibeglogene autotemcel/ß-thalassemia
Carvykti	Janssen Biotech	2022.02	Gene Therapy	CAR-T/multiple myeloma
Rethymic	Enzyvant Therapeutics	2021.08	Tissue-engineered therapy	Thymus tissue-agdc/congenital athymia
Stratagraft	Stratatech	2021.06	Tissue-engineered therapy	Keratinocytes and dermal firoblast in collagen/debrided thermal burns
Abecma	Celgene	2021.03	Gene Therapy	CAR-T/Multiple myeloma
Breyanzi	Juno Therapeutics	2021.02	Gene Therapy	CAR-T/B cell Lymphoma
Tecartus	Kite Pharma	2020.07	Gene Therapy	CAR-T/Acute Lymphoblastic Leukemia

*Modified from the U.S. FOOD & DRUG ADMINISTRATION (U.S., Food & drug administration, 2024).

This evolving field of biology is growing and changing so fast that it is challenging for the regulatory bodies to keep pace. Countries have enacted legislation for RM, and the global regulatory bodies have published a series of guidance documents outlining steps to protect consumers against the potential dangers of unproven treatments while ensuring quicker access to patient benefits. In this review, we briefly summarize global legislation on regenerative medicine (RM), which serves as the supreme law for multiple guidelines. We examine the main regulatory authorities responsible for evaluating Investigational New Drug (IND) applications. The key features of RM legislation in Asia, the United States, and the European Union, with a particular focus on the approval processes, are described. Additionally, recent developments in RM legislation across these regions are discussed.

### Legislations and regulations for RM world-wide

In September 2014, the Japanese Diet, or legislature, enacted a set of laws and amendments intended to improve and simplify the regulation of regenerative medicine in Japan (Konomi et al., 2015; Azuma and Yamanaka, 2016; Sipp and Okano, 2018). Following this, other countries in Asia, including South Korea and Taiwan, have established legal frameworks and regulatory guidelines for regenerative medicine, reflecting the rapid advancements in this field. Over the last decade, as the field of RM has evolved, RM legislation has been promoted and strengthened. In this review, we examine RM legislation in the US, EU, and Asian countries, providing a global comparison of these legislative frameworks (Table 2).

**TABLE 2:** The global regulatory framework for RM.

Country	Legislation	^a^Regulatory body	Review board	Key features
United States	21st Century Cures Act	FDA	Center for Biologics Evaluation and Research (CBER)	Expedited pathways (RMAT) for cell therapies, gene therapies; focus on advanced therapies
European Union	Regulation (EC) No 1394/2007	EMA	Committee for Advanced Therapies (CAT)	Centralized marketing authorization for gene therapies, cell therapies, and tissue-engineered products
Canada	Food and Drugs Act	Health Canada	The Biologic and Radiopharmaceutical Drugs Directorate (BRDD) under Health Canada	Regulates as biologics; thorough review of quality, safety, and efficacy for market approval
United Kingdom	Human Medicines Regulations 2012 The Human Tissue (Quality and Safety for Human application) Regulations 2007	MHRA	MHRA	Continues EU-based regulations for ATMPs, with centralized approval process maintained post-Brexit
Japan	Act on the Safety of Regenerative Medicine Act Pharmaceuticals and Medical Devices Act	PMDA MHLW	Certified Regenerative Medicine Committee, CRMC	Fast-track approval system based on demonstrated safety and probable efficacy, followed by post-marketing surveillance
South Korea	Pharmaceutical Affairs Act Advanced Biomedical Regeneration Act	MFDS MOHW	Advanced Regenerative Medicine and Advanced Biological Products Policy Review Committee	Specific regulations including expedited review for critical therapies; detailed safety monitoring and governance
Taiwan	Regenerative Medicine Act	TFDA	Regenerative Medicine Review Committee	Referenced to Japan and Korea’s management system. RMA implemented conditional and time-limited (CTL) approval regulatory framework similar to Japan’s PMD Act

^a^ FDA, U.S., food and drug administration; EMA, european medicines agency; MHRA, medicines and healthcare products regulatory agency; PMDA, pharmaceuticals and medical devices agency; MHLW, ministry of health, Labour, and Welfare; MFDS, ministry of food and drug safety; MOHW, ministry of health and welfare; TFDA, taiwan food and drug administration.

#### United States

In 2016, 21st Century Cures Act^1^ brought significant changes to RM regulation. It aimed to improve The U.S. Food and Drug Administration (FDA) regulation, support, and accelerate the innovation of medical technology to the market (Barlas, 2018). Specifically, it introduced the Regenerative Medicine Advanced Therapy (RMAT) designation to expedite the development and review of regenerative medicine products (Section 3033 of the 21st Century Cures Act) (ADMINISTRATION USFD, 2023) The drug product of regenerative medicine therapy is defined as a cell therapy, therapeutic tissue engineering product, human cell and tissue product, or any combination product using such therapies or products, except for those regulated solely under Section 361 of the Public Health Service Act and Part 1,271 of Title 21, Code of Federal Regulations. Cellular therapy and tissue engineering products are regulated under FDA regulations, specifically outlined in 21 CFR Part 1,271 (11) Manufacture of human cells, tissues, and cellular and tissue-based products (HCT/Ps) requires the establishment of donor eligibility, current good tissue practices, and other procedures to prevent the introduction, transmission, and spread of communicable diseases by HCT/Ps. The 21 CFR Part 1,271 includes requirements for the collection, processing, storage, transplantation, and use of human cells, tissues, and cellular therapy products. The FDA has specific guidelines and pathways for the approval of regenerative medicine products, including the Regenerative Medicine Advanced Therapy (RMAT) designation from 2017. In that year, the FDA granted approval for the first regenerative medicine product targeting spinal cord injuries, highlighting advancements in therapeutic applications. The FDA has engaged RM-specific committees composed of stakeholders to address the scientific, medical, and regulatory challenges posed by these therapies and has reorganized the Center for Biologics Evaluation and Research (CBER) (21 USC 399g, Sec. 1014. FOOD AND DRUG ADMINISTRATION INTERCENTER INSTITUTES)^1^ (CBER, 2018). The Center for Biologics Evaluation and Research (CBER) regulates cellular therapy products, human gene therapy products, and certain devices related to cell and gene therapy. CBER utilizes the Public Health Service Act and the Federal Food, Drug, and Cosmetic Act as the foundational legislation for its oversight functions. The regulation of cellular and gene therapy products falls under the responsibility of the Office of Tissues and Advanced Therapies (OTAT/CBER/FDA) within CBER (Lapteva et al., 2018; Qiu et al., 2020).

#### Canada

In Canada, the regulatory framework for drugs is primarily overseen by Health Canada, which ensures that therapies and products meet safety, efficacy, and quality standards. Although regenerative medicine is not specifically listed in the Food & Drug Act in Canada^2^, Food & Drug Act provide legal authority to Health Canada to regulate and oversee development and marketing of RM (Chisholm et al., 2019). Under the Food and Drugs Act, RM products can be regulated by several regulations, including the Food and Drug Regulations, Processing and Distribution of Semen for Assisted Conception Regulations, Safety of Human Cells, Tissues and Organs for Transplantation Regulations, Medical Devices Regulations, and Natural Health Products Regulations (Chisholm et al., 2019; Viswanathan and Bubela, 2015). The Biologic and Radiopharmaceutical Drugs Directorate (BRDD) within Health Canada is responsible for the review and approval of RM (Canada, 2021; Canada Go, 2020). Although, there is no formal regulatory definition of regenerative medicine directly in Canada, and cell therapies are not specifically listed in the Food & Drugs Act (Chisholm et al., 2019), the Food and Drugs Act and its associated regulations classify regenerative medicine products under biologic drugs, requiring rigorous clinical trials and evaluations for market authorization (Chisholm et al., 2019; Don Husereau SRA et al., 2021).

#### European Union

The law that defines and regulates Advanced Therapy Medicinal Products (ATMPs) in the European Union is Regulation (EC) No 1394/2007 (PaMD, 2007)^3^ and Directive 2009/120/EC amending Directive 2001/83/EC^4^ relating to medicinal products for human use as regards advanced therapy medicinal products. Regulation (EC) No 1394/2007 was established in 2007 and came into forced on 30 December 2008, to provide a comprehensive framework for the regulation of “advanced therapy medicinal products” in EU. At the European level, it sets out clear rules and regulations governing ATMPs products across the European Union member States. This regulation covers a range of ATMPs, including gene therapy products, cell therapy products, tissue-engineered products and, some combined ATMPs which may contain one or more medical devices as an integral part of the medicine (Agency EM, 2016). It establishes requirements for the ATMPs authorization. Later, the definitions and detailed scientific and technical requirements for advanced therapies were updated in directive 2009/120/EC (Annex PART IV, 2) (AGENCY, 2000). It also established detailed scientific and technical requirements for tissue-engineered products, as well as for ATMPs containing devices and combined ATMPs (Annex I to Directive 2001/83/EC). For the ATMP product authorization and supervision, the European Medicines Agency (EMA) evaluate the regenerative medicines within the EU through the Advanced Therapy Medicinal Products (ATMP) regulations. Under the Regulation (EC) No 1394/2007^3^, a Committee for Advanced Therapies (CAT) within the EMA was established (AGENCY, 2000). CAT is responsible for preparing a draft opinion on the quality, safety and efficacy of each ATMP (Article 215, Regulation (EC) No 1394/2007) before the Committee for Medicinal Products for Human Use (CHMP) in EMA adopts a final opinion on the marketing authorization of the medicine concerned (AGENCY EM, 2015).

#### United Kingdom

The UK is establishing its regulatory pathways, continuing to regulate advanced therapy medicinal products (ATMPs) under the guidelines that were in place during its membership in the EU, managed by the Medicines and Healthcare products Regulatory Agency (MHRA). The definition of ATMPs is found in the Human Medicines Regulations 2012^5^ (PART1, 2A), which includes a gene therapy medicinal product, a somatic cell therapy medicinal product, and a tissue engineered product. The Human Medicines Regulation is applicable to the products for sale or supply in Great Britain only (PART1, 2A). The combined advanced therapy medicinal product is defined as an advanced therapy medicinal product which incorporates, as an integral part of the product, one or more medical devices or one or more active implantable medical devices; and the cellular part (PART1, 2A). The MHRA is the competent authority for all medicinal ATMP products, including combination ATMPs. If tissues and cells are being used as starting materials in a medicinal product, the donation, procurement and testing of the cells are covered by The Human Tissue (Quality and Safety for Human application) (Ikka et al, 2023)^6^.

#### Japan

In November 2014, Japan enacted the Regenerative Medicine Promotion Act. Then, two other pieces of legislation for RM were enacted. The first one was The Act on the Safety of Regenerative Medicine (ASRM)^7^, and the second one was the Pharmaceuticals, Medical Devices, and Other Therapeutic Products Act (PMD Act)^8^. The ASRM applies to medical treatments that intend to use cell-processed products (Article 2). Under the ASRM Act, clinical research, other than clinical trials intended for submission to gain approval or daily medical treatments that use unapproved regenerative medicine products, is covered (Azuma, 2015). The applicable clinical trials are reviewed by certified committees for RM (Certified Regenerative Medicine Committee, CRMC) or the Certified Special Committee for Regenerative Medicine accredited by the Ministry of Health, Labour, and Welfare (MHLW) (Articles 26–34) (Ikka et al., 2023; Tobita et al., 2016). CRMC approves and regulates cell-processing facilities and quality standards to ensure the safe use of stem cell-based therapy (Azuma, 2015). Notably, the ASRM excludes stem cell-based therapy being developed for or entering the pharmaceutical market, indicating that it is for clinical trials rather than product approval (Takashima et al., 2021). On the other hand, the PMD Act approves stem cell-based drug products (Article 14). The PMD Act regulates the commercialization of regenerative medicinal products, which are defined as processed (more than minimal manipulation) live human/animal cells intended to be used for the reconstruction, repair, or formation of structures or functions of the human body (Takashima et al., 2021). It includes gene therapy (PaMD). The PMD Act introduces a conditional and time-limited approval system for regenerative medical products, allowing these therapies to reach the market more quickly (Articles 23–26, The Pharmaceutical and Medical Devices Act). Products can be conditionally approved based on preliminary safety and efficacy data, with full approval contingent upon further validation (Matsushita et al., 2020). In Japan, the Pharmaceuticals and Medical Devices Agency (PMDA) is the regulatory body for regenerative medicine product approval. For marketing authorization, PMDA receives the applications and evaluates the product’s quality, safety, and efficacy, performs a risk assessment, and recommends approval through either the full approval pathway or the conditional time-limited approval pathway (PMD Act)^8^. The conditional approval pathway is typically reserved for products where safety is confirmed but efficacy data are insufficient (Konomi et al., 2015; Matsushita et al., 2020).

#### South Korea

The legislative framework for regenerative medicine in South Korea is primarily governed by the Act on the Safety and Support for Advanced Regenerative Medicine and Advanced Biopharmaceuticals (ARMAB)^9^, enacted in 2019 and implemented in 2020 by Ministry Of Health and Welfare (MOHW) (Kim and Bae, 2022). This act aims to promote applications of RM while ensuring patient safety by establishing comprehensive guidelines for the quality, safety, and efficacy of regenerative medicine products (Article 1). South Korea’s ARMAB typically establishes Advanced Regenerative Medicine Safety Management Institutions under MOHW to ensure the safety of RM and is responsible for the management and supervision of RM institutes, and the establishment of a follow-up system to investigate the causes of adverse reactions after clinical research of advanced regenerative medicine (Article 19). ARMAB also established the Advanced Regenerative Medicine and Advanced Biopharmaceuticals Review Committee, which approves clinical studies by MOHW. Under the ARMAB Act, the committee reviews the clinical research plan, manages the long-term follow-ups, and designates institutes of advanced regenerative medicine application for clinical studies and cell processing (Article 12). Before the ARMAB legislation enacted, Pharmaceutical Affairs Act^10^ and Regulation on Review and Authorization of Biological Products^11^ the Pharmaceutical Affairs Act and Regulation on Review and Authorization of Biological Products from the Korea Ministry of Food and Drug Safety (MFDS) primarily governed the approval and regulation of regenerative medicine, including cell therapy, gene therapy, and combined therapy. Similar to the Japanese ASRM, clinical studies other than clinical trials or daily medical treatments that use unapproved regenerative medicine can be approved by ARMAB. In fact, only clinical studies for regenerative medicine can be applied to the ARMAB track, except for clinical trials. Clinical trials can be applied to the MFDS track only. As of 2024, ARMAB was revised so that the term “treatment” is newly included. A patient who has never been involved in a clinical study can be treated with the approved therapy, although the investigator cannot request a fee from the patient (Article 10). (Safety MoHaWAMoFaD) For the RM drug product clinical trial and product approval, MFDS reviews the application under the Pharmaceutical Affairs Act and regulations.

#### Taiwan

RM is regulated by the Taiwan Food and Drug Administration (TFDA), established in 2010, as “medicinal products” under the Pharmaceutical Affairs Act. on 4 June 2024, Taiwan’s Legislative Yuan passed two drafts of regenerative medicine legislation proposed by the Ministry of Health and Welfare, Taiwan. The Regenerative Medicine Act (RMA)^12^” and the Regenerative Medicine Product Regulations. The Regenerative Medicine Acts were promulgated by the President on 19 June 2024, officially establishing a regulatory framework for regenerative medicine technologies and pharmaceuticals at the legal level, outlining a foundation for the development of Taiwan’s regenerative medicine industry. In the RMA, regenerative medicine is defined as the use of genes, cells, and their derivatives to treat, repair, or replace human cells, tissues, and organs (Article 3)^12^. The RMA references Japan’s and Korea’s management systems, leading to the formulation of management regulations for regenerative medicine implementation. The RMA implemented a conditional and time-limited (CTL) approval regulatory framework, similar to Japan’s PMD Act, to expedite the availability of these therapies for patients with serious or life-threatening conditions. Before CTL approval by the Regenerative Medicine Review Committee, the central competent authority must grant approval (Article 6). The Ministry of Health and Welfare (MoHW) reviews and approves the application based on a risk-benefit analysis, requiring ongoing studies and data submission to confirm long-term safety and effectiveness. The long-term safety study is a part of regulatory requirements in Japan, South Korea, and Taiwan.

## Discussion

### Current changes on regenerative medicine legislations

As the regenerative medicine therapies have emerged, it challenges the existing regulatory structure to be reformed or newly established.

In the US, FDA has published four guidance documents^13^ ^,^ ^14^ ^,^ ^15^ ^,^ ^16^ that are part of a comprehensive policy framework to address changes of RMAT products including human cells, tissues, and cellular and tissue-based products. The guidelines provided a flexible regulatory framework for the newly developed RM and helps the innovative treatment options to the patients.

In the EU, The Congress modernizing the EU legislation on ATMPs. In April 2023, the European Commission (EC) published two proposals to revise the existing (and outdated) pharmaceutical legislation (https://health.ec.europa.eu/medicinal-products/pharmaceutical-strategy-europe/reform-eu-pharmaceutical-legislation_en). The stakeholders of ATMPs recommended to reform of the Hospital Exemption (HE) scheme for Advanced Therapy Medicinal Products (ATMPs) in the legislation. The current ATMP Regulation (EC) No 1394/2007, Article 28 (2) introduces the Hospital Exemption (HE) scheme, allowing Member States to permit the use of an ATMP without marketing authorization under specific conditions. Due to the inconsistent interpretations about the HE across Member States, stakeholders recommended to clarify the scope of the HE scheme, ensuring it remains an exception for cases where no authorized or trial alternatives exist, and enhancing data transparency and safety measures.

In Asia, the new potential therapies have prodded Congress to make the pathway to the marketplace. Taiwan enacted laws as of 2024 June that Legislative Yuan passed the Regenerative Medicine Treatments Act and the Regenerative Medicinal Products Act. In Korea MOHW enacted legislation on RM as of 2019 which revised currently to improve accessibility of the patient^10^.

### Key features of legislations for Asian country (Japan, Korea, Taiwan) and US/EU

There are key features of legislation in the three Asian countries compared to US/EU legislation. First, the Ministry of Health, Labor and Welfare (MHLW) in Japan, Ministry of Health and Welfare (MOHW) in Korea, and Ministry of Health and Welfare (MOHW) in Taiwan proposed acts for regenerative medicine and approve clinical studies. The government supports dual-tracks of the regulatory pathway, aiming to improve accessibility and flexibility for clinical operations while ensuring safety by monitoring post-treatment. Second, the law implements a risk-based classification system for cell-based interventions and provides for a review of research and therapeutic plans for regenerative medical procedures by authorized committees. Third, the definition of RM products in the three Asian countries follows the EU, where RM products are classified into four categories: gene therapy products, cell therapy products, tissue-engineered products, and composite products, rather than RMAT, which is defined as a cell therapy, therapeutic tissue engineering product, human cell and tissue product, or any combination product.

### The need for global harmonization in regenerative medicine regulations

The governmental oversight and regulation will remain important for the future of regenerative medicine and the application of safe and effective cellular treatments for patients. Therefore, we need a global consensus on scientific disciplines, so-called regulatory science, for better decision-making throughout the quality, safety, and efficacy assessment of RM globally. In this review, we reviewed RM legislation and regulatory bodies and committees in each country: the US, Canada, EU, UK, Japan, Korea, and Taiwan. We found that the development of legislation in each country affects each other’s regulatory pathway structures and contents. Inconsistency in regulatory requirements across countries remains a major hurdle for manufacturers and patients to progress and access these treatments harmoniously. For example, Tisagenlecleucel (Kymriah^®^) was the first cell-based gene therapy approved by the US FDA and in 34 other countries (Awasthi et al., 2023), yet it is not approved in Scotland. From this point of view, we need a comprehensive comparison of lessons learned from each other’s legislation and a focus on efforts toward global harmonization, especially for those countries drafting RM-specific legislation. This review offers a brief overview of global RM legislation and the relevant regulatory authorities. For the development of practical guidelines grounded in international consensus, a more thorough and comprehensive analysis of the specific provisions within each legislative framework is necessary.

## Footnotes

1 21st Century Cures Act https://www.congress.gov/114/plaws/publ255/PLAW-114publ255.pdf.

2 Food and Drugs Act (R.S.C., 1985, c. F-27) https://laws.justice.gc.ca/eng/acts/f-27/page-1.html.

3 Regulation (EC) No 1394/2007 on Advanced Therapy Medicinal Products https://eur-lex.europa.eu/legal-content/en/ALL/?uri=CELEX%3A32007R1394.

4 Directive 2001/83/EC of the European Parliament and of the Council https://eur-lex.europa.eu/legal-content/EN/ALL/?uri=celex:32001L0083.

5 The Human Medicines Regulations 2012 https://www.legislation.gov.uk/uksi/2012/1916/part/1.

6 The Act on the Safety of Regenerative Medicine (ASRM) https://laws.e-gov.go.jp/law/425AC0000000085.

7 G. Pharmaceuticals, Medical Devices, and Other Therapeutic Products Act (PMD Act) https://www.japaneselawtranslation.go.jp/en/laws/view/3213.

8 H. Medical Practitioners’ Act https://www.japaneselawtranslation.go.jp/en/laws/view/3992.

9 Act on the Safety and Support for Advanced Regenerative Medicine and Advanced Biopharmaceuticals https://elaw.klri.re.kr/eng_mobile/viewer.do?hseq=56486&type=part&key=37.

10 Pharmaceutical Affairs Act https://elaw.klri.re.kr/eng_service/lawView.do?lang=ENG&hseq=62649.

11 Regulation on Approval and Review of Biological Products https://www.mfds.go.kr/eng/brd/m_27/view.do?seq=71480&srchFr=&srchTo=&srchWord=&srchTp=&itm_seq_1=0&itm_seq_2=0&multi_itm_seq=0&company_cd=&company_nm=&page=1.

12 Regenerative Medicine Act (RMA) Order No. 11300054291 https://law.moj.gov.tw/News/NewsDetail.aspx?msgid=184596.

13 Regulatory Considerations for Human Cell, Tissues, and Cellular and Tissue-Based Products: Minimal Manipulation and Homologous Use https://www.fda.gov/regulatory-information/search-fda-guidance-documents/regulatory considerations-human-cells-tissues-and-cellular-and-tissue-based-products-minimal.

14 Same Surgical Procedure Exception: Questions and Answers Regarding the Scope of the Exception https://www.fda.gov/regulatory-information/search-fda-guidance-documents/same-surgical-procedure-exception-under-21-cfr-127115b-questions-and-answers-regarding-scope.

15 Expedited Programs for Regenerative Medicine Therapies for Serious Conditions https://www.fda.gov/regulatory-information/search-fda-guidance-documents/expedited-programs-regenerative-medicine-therapies-serious-conditions.

16 Evaluation of Devices Used with Regenerative Medicine Advanced Therapies https://www.fda.gov/regulatory-information/search-fda-guidance-documents/evaluation-devices-used-regenerative-medicine-advanced-therapies.
